# Recursive bit assignment with neural reference adaptive step (RNA) MPPT algorithm for photovoltaic system

**DOI:** 10.1038/s41598-023-28982-6

**Published:** 2023-03-14

**Authors:** Eman Hegazy, Mona Shokair, Waleed Saad

**Affiliations:** 1 Department of Electrical and Electronic Engineering, Faculty of Electronic Engineering, Menoufia University, Menouf, 32951 Egypt; 2 Department of Electrical Engineering, College of Engineering, Shaqra University, 75311 Dawadmi, Saudi Arabia

**Keywords:** Energy harvesting, Renewable energy

## Abstract

Recent research has focused on photovoltaic (PV) systems due to their important properties. The efficiency of the PV system can be enhanced by many Maximum Power Point Tracking (MPPT) algorithms proposals. MPPT algorithms are used to achieve maximum PV output power by optimizing the duty cycle of the DC–DC buck/boost converter. This paper introduces an RNA algorithm as an efficient MPPT algorithm for the photovoltaic system. This proposed RNA algorithm consists of two main segments. The first segment is an artificial neural network for generating reference power. The second segment is a proposed Recursive Bit Assignment (RBA) network to allow variable step size of the boost converter duty cycle. The instant PV power adopts the RBA network to produce the variable duty cycle increment value. Additionally, the neural network is implemented in such a way to obtain the best performance. Many simulation results using MATLAB to test the system performance are presented. The performance characteristics of the photovoltaic system with variable irradiance and variable temperature are simulated. From results, the proposed RNA algorithm achieves fast tracking time, high energy efficiency, true maximum power point and acceptable ripple. Additionally, comparisons between the RNA algorithm and other related algorithms such as Perturb and Observe, the Neural Network and the Adaptive Neural Inference System Algorithms are executed. The proposed RNA algorithm achieves the best performance in all case studies such as; irradiance profile variation, severe temperature and irradiance diversions, and partial shading conditions. Besides, the experimental circuit of the PV system is also presented.

## Introduction

Nowadays, solar photovoltaic (PV) is getting a lot of attention in the field of renewable energy. Photovoltaic solar energy harvesting systems have been widely used in many high and low energy applications for example in wireless sensor networks. The photovoltaic module is usually followed by the DC-DC boost converter to adapt stable output voltage to the load. While the main challenge of the PV module is its low efficiency, as it is in the range of 10–40%^1^. Moreover, the maximum output power of the PV module changes under the changing environmental conditions.

Therefore, the MPPT control circuits are used. It controls the operation of the DC-DC boost converter circuit to keep track of the maximum value of the PV output power to get maximum efficiency. There are several MPPT algorithms to get the maximum power point (MPP) from the PV module such as perturbation and observation (P &O) technique, the incremental conductance (IncCond) technique, ripple correlation technique, short circuit current (SCC) technique, open circuit voltage (OCV) technique, etc.^2,3^.

This paper presents a proposed two-stage RNA algorithm which are NN stage and RBA stage. In the first stage of the proposed system, NN is used because in recent years neural networks have become more attractive for their ability to solve nonlinear and complex problems. In it, the values of irradiation (G) and temperature (T) are entered into the trained NN model to calculate the equivalent voltage and corresponding current of the PV module. In the second stage of the proposed system, a proposed circuit named RBA is implemented to obtain the value of the variable step size of the DC-DC boost converter. Extensive MATLAB simulation programs have been implemented to study the proposed system. Moreover, many comparisons have been made with other MPPT algorithms such as P &O, NN and ANFIS algorithms. It has been found through the results that the proposed algorithm achieves the fastest rise time with high power efficiency, while working with slightly higher ripple.

The objective of this paper is to present an efficient MPPT control algorithm to improve the performance of the PV system. Moreover, the performance of the entire system is verified. The main contributions of this paper can be summarized as follows:

- Proposing an efficient MPPT control algorithm called “RNA” based on neural network and the proposed RBA network.
- Design an efficient RBA block for evaluating the variable step size of the boost converter circuit.
- Design an efficient neural network based on the measured input irradiance and temperature to simulate the PV module.
- Evaluating and compare the performance of the proposed RNA algorithm with other relevant MPPT algorithms.
- Testing the performance of the proposed system under severe shaded conditions
- Studying the effect of sampling time on system performance.
- Studying the impact of fast temperature changes on system performance with different irradiance levels at the same time.
- Experimentally implementation the proposed RNA algorithm to verify the simulation results.

The rest of the paper is organized as follows: “Related work” section presents the related work. The whole PV system architecture description is shown in “System description” section. In the “Proposed model” section, the proposed RNA algorithm exists in detail. Then, the performance metrics are explained in “Performance metrics” section. After that, the simulation analysis is provided in the “Simulation analysis” section. Finally, the conclusions and future work remarks are shown in the “Conclusions” section.

## Related work

Many algorithms are evolved to extract the Maximum Power Point (MPP) from the solar PV module under varying conditions of solar irradiance and temperature^4^. The traditional techniques of MPPT algorithms, which are based on the hill-climbing method such as Perturbation and Observation (P &O), Incremental Conductance (INC), Fractional Open Circuit Voltage(OCV), and Fractional Short Circuit Current techniques, have been discussed in^5–11^. These algorithms present a slow response under varying irradiation levels. The heuristics-based algorithms like particle swarm optimization (PSO), Firefly Algorithm (FA), and genetic algorithm (GA) have been released to improve the MPPT performance under both partial shading, and uniform irradiance climatic conditions as in^12–14^.

one of the most traditional techniques is P &O which has been redeveloped in^15^ to extract the Global Maximum Power Point (GMPP) among the multiple Local Maximum Power Point (LMPP) in the PV characteristics curve under Partial Shading Conditions (PSCs). This can be done by utilizing Trapezoidal concept in the GMPPT tracking process. This proposed concept using P &O has been performed under uniform irradiance and step variations in irradiance levels. The proposed technique can achieve a best tracking time less than 100 ms with a reduced steady-state oscillation. An improved adaptive step size P &O has been proposed in^16^ to attain the maximum power point of the solar PV under different weather conditions with a low power oscillation. Also, the lead-acid battery station can be charged using a three-stage charging controller (TSCC) as a battery charging control unit. The proposed technique has been scored a best results compared with the conventional P &O in terms of a half power losses and a minimized oscillations around MPP.

Due to the main defect of using fixed step size MPPT algorithms like P &O and INC, the authors in^17^ have been presented an adaptive MPPT algorithm using a variable step size to improve the PV efficiency. The proposed algorithm introduced many advantages such as fast dynamic response, a low oscillations, and fast tracking speed under different weather conditions. A new MPPT algorithm for the PV system has been suggested in^18^ in order to extract the actual MPP under different weather conditions. It Also called an Adjustable Step Size Theta Approach (ASSTA). The novel approach has been achieved a fast tracking speed to the actual MPP uner a rapid changing in the weather conditions.

The recent techniques such as artificial neural network^19–21^, fuzzy logic methods^22,23^, and artificial neuro-fuzzy inference system^24,25^. The artificial neural techniques have become the most interesting approaches that are commonly used in PV systems due to their ability to resolve significant problems of the traditional methods such as oscillation around the maximum point and failure behavior with rapid changing of the solar irradiance. Several MPPT controllers based on the artificial neural network have been developed due to the main advantages of the neural network, such as it can find complex nonlinear relations between the independent and dependent variables without the need for an accurate mathematical models.

An artificial neural network has been introduced for fast-tracking of maximum power point of the solar PV as in^26^ . In this algorithm, the output of the neural network is the reference voltage of the MPP of the solar PV under different climatic conditions. The authors have been presented an efficient artificial neural network-based MPPT scheme for improving the efficiency of the photovoltaic generator. It can be operated accurately and rapidly at MPP without power loss. This can be done through matching impedance between solar PV module and load by using a DC-DC boost converter in which its duty cycle is set by artificial NN.

Increasing the effectiveness of the solar PV systems can be done by improving the PV panel efficiency. Several researchers have been utilized the Artificial Neural Network (ANN) as an intelligent algorithm to donate a fast tracking of MPP, a fewer oscillations, and an improved performance of 98%^27^. In this article, many algorithms based on ANN or a hybrid combination with fuzzy or a meta heuristic algorithm have been presented.

The authors have been proposed two artificial neural network-based MPPT controllers: fixed-step and variable step NN^28^. The neural network-based MPPT controller is executed in two phases: online and offline. To find the optimal NN, a different set of neural network parameters must be trained through the offline step. The optimal neural network-based MPPT controller can be used in the PV system through the online step. In^29^, the authors have been investigated a low complexity MPPT algorithm based on the neural network model of the solar PV. The expression for the output current of the NN model can explore a gradient, analytical MPPT method which can donate an accurate prediction of the maximum power.

The researchers in^30^ presented a neural network assisted variable step size VSS incremental conductance as a MPPT technique. The main role of the neural network is to define an optimal scaling factor that must be utilized in the current irradiance level for VSS incremental conductance MPPT method. Hence, the performance of the traditional VSS conductance has been mended with a rapid changes in the irradiance levels.

The proposed algorithm has lower computational complexity than the other NNs based MPPT techniques as the position of the MPP is determined by one multiplayer NN or by using two single layers NNs. An adaptive neuro-fuzzy system-based MPPT algorithm with PI controller has been provided in^31^. The proposed algorithm donates a maximum power point tracker with a control gain for the solar pumping system. The proposed technique in^32^ improved the efficiency of the solar PV harvesting system by minimizing the energy losses caused by the MPPT controller and dc-dc converter. This can be done by using a successive approximation register-based MPPT algorithm. This algorithm has two main benefits over the other MPPT algorithms in energy savings and power consumption. It is considered an advanced version of the hill-climbing technique fast-tracking time. A maximum power point tracking circuit that used an analog to digital converter with a 4-bit successive approximation register for solar PV harvesting system has been suggested in^33^. This circuit achieves high MPPT efficiency with low power consumption.

## System description

**Figure 1:**
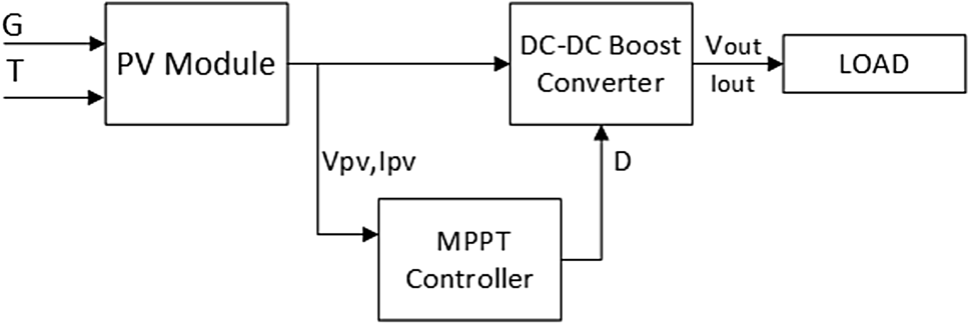
The solar PV harvesting system architecture.

**Figure 2:**
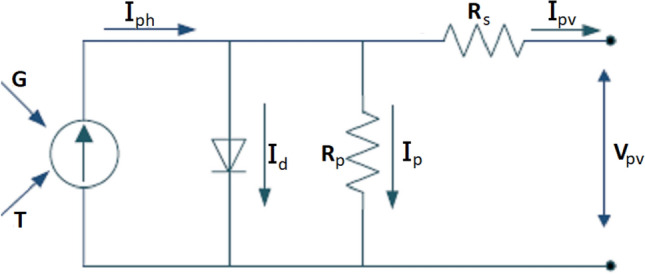
The equivalent circuit model of the PV module.

The architecture of the solar PV system consists of a PV module, a DC-DC boost converter, an MPPT control circuit, and a load. The main challenge for this system is the maximum power tracking process under different environmental conditions such as G and T. The system architecture is plotted in Fig. 1. The equivalent circuit of the solar PV module can be modeled as a single diode circuit. The mathematical expression for the nonlinear I–V characteristics of the ideal solar PV cell is described as follows^24^:

1 $$\begin{aligned} {{I_{pv}}={{I}_{ph}}-{{I}_{o}}\left[ \exp \left( \frac{qv}{aKT} \right) -1 \right] } \end{aligned}$$

where $I_{Ph}$ is the photocurrent (generated current of the incident light) which is a function of the solar radiation (G). $I_o$ is the reverse saturation current. q is charge of electron ($1.6\times {{10}^{-19}}$ c). *v* is the open-circuit voltage. K is the Boltzmann constant ($1.38\times 10{{-}^{19}}$ J/K), *T* is the temperature of the solar cell (300 kelvin), and *a* is diode ideality constant. Practically, the equivalent circuit of the solar PV is with two shunt and series resistances ($R_s$ and $R_p$) as shown in Fig. 2. The output current of the practical PV module $I_{pv}$ can be expressed as^26^:

2 $$\begin{aligned} { {I_{pv}}={{I}_{ph}}-{{I}_{o}}\left[ \exp \left( \frac{q(V_{pv}+{I_{pv}}{{R}_{s}})}{aKT} \right) \right] -\left( \frac{V_{pv}+{I_{pv}}{{R}_{s}}}{{{R}_{p}}} \right) } \end{aligned}$$

where $$V_{pv} $$ is the output PV voltage. $$R_{p}$$ is the shunt resistance. $$R_{s} $$ is the series resistance.

## The proposed recursive bit assignment with neural reference adaptive step (RNA) algorithm

The proposed RNA algorithm consists of two main steps. First, the feed-forward neural network is used. It has two inputs which are the radiation G and the temperature T. Hence, the maximum power point $$P_{mpp} $$ is the output of the neural network. It can be used as a reference power for the second step. The second step is the recursive bit allocation used to obtain the adaptive duty cycle.

### The proposed RNA architecture

**Figure 3:**
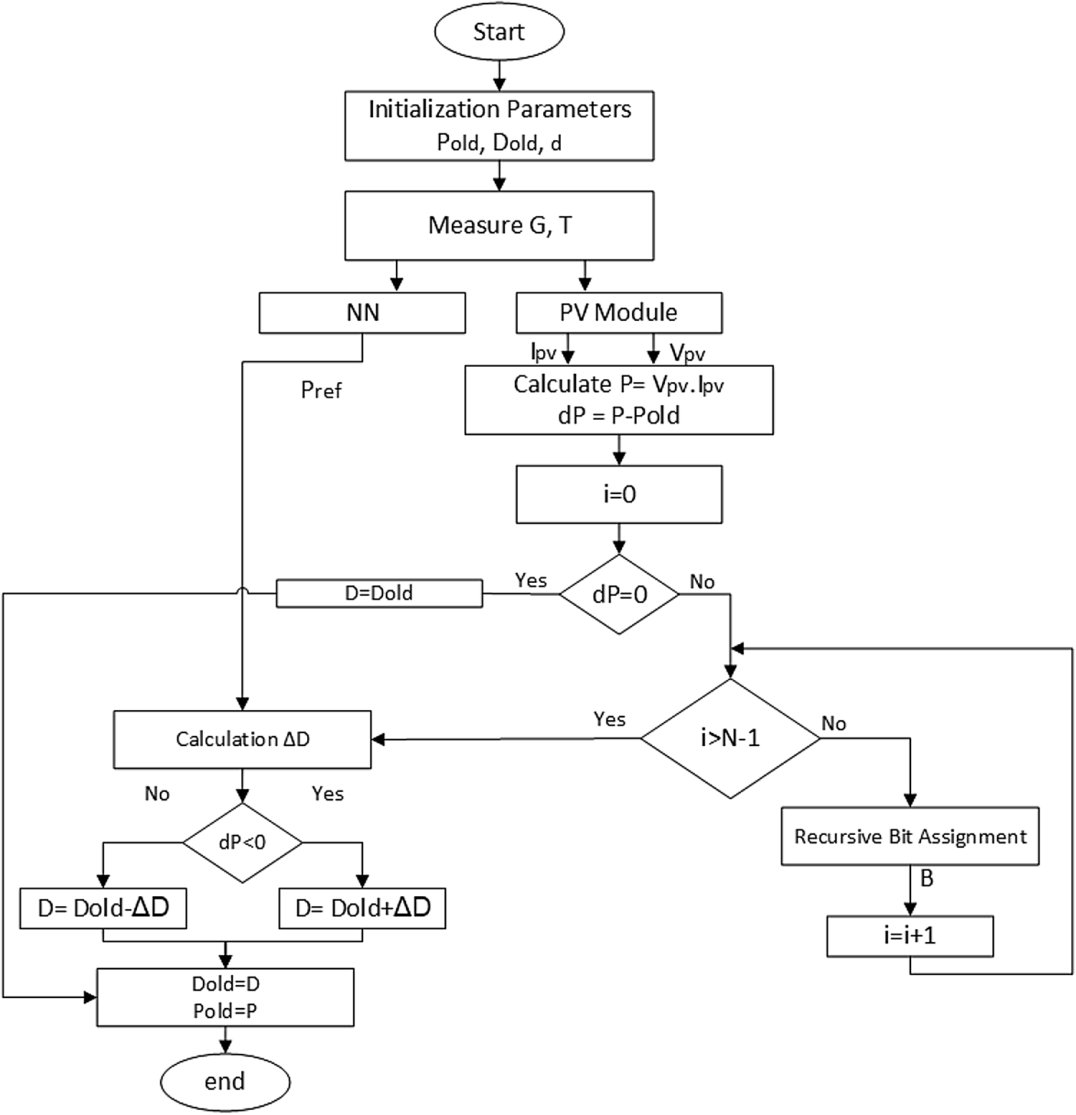
The flowchart of the proposed RNA algorithm.

The flowchart of the proposed algorithm is shown in Fig. 3. It can be explained as follows: Firstly, the initialization parameters which are the initial value of power ($P_{old} =0$), the initial value of the duty cycle ($D_{old}$) and the fixed step size ($d= 0.00001$) are set. $D_{old}$ can be donated as follows:

3 $$\begin{aligned} D_{old}= D_{min} + rand* [ D_{max} - D_{min}] \end{aligned}$$

where $D_{max}$ is the maximum duty ratio (equals 0.8), $D_{min}$ is the minimum duty ratio (equal 0.08), and *rand* is a random value from 0 to 1.

**Figure 4:**
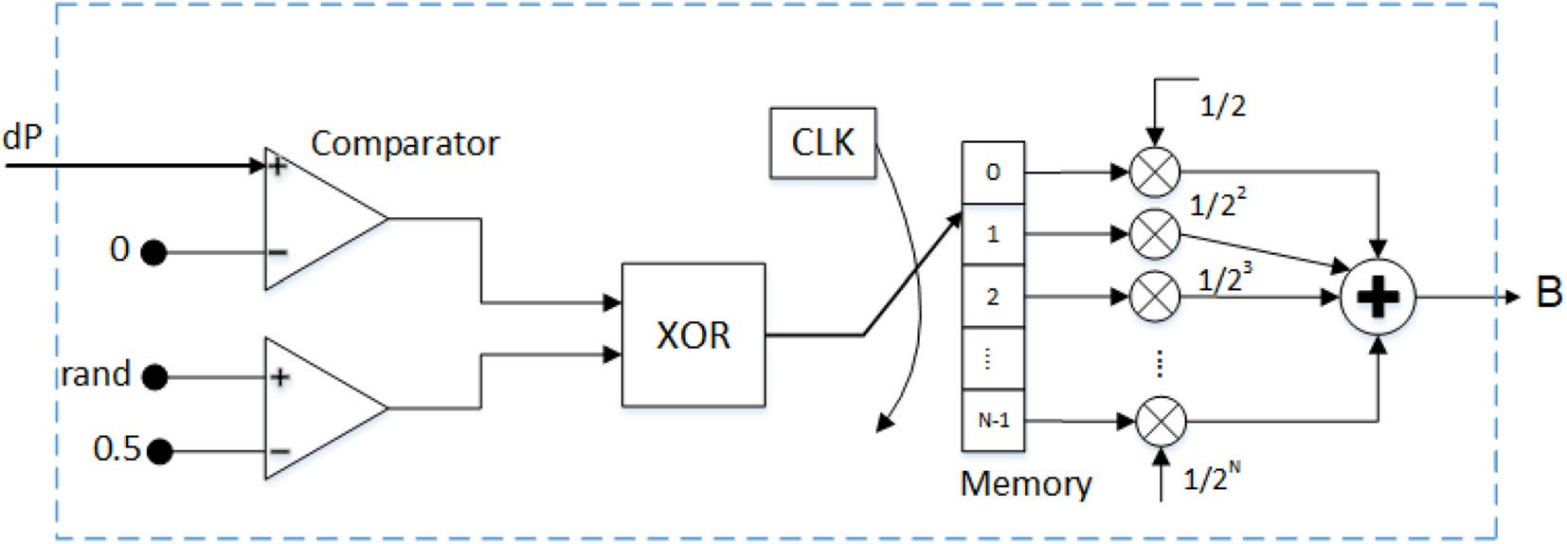
The recursive bit assignment (RBA) block.

Then, the irradiance (G) and the temperature (T) are measured. These values are used as inputs for the PV and the NN modules. The outputs of the PV module are the voltage ($V_{pv}$) and current ($I_{pv}$) of the PV. After that, the PV power can be calculated by multiplying ($V_{pv}$) and ($I_{pv}$). Hence, the power difference (*dP*) can be estimated by subtracting the $P_{old}$ from the instantaneous PV power value. Thereafter, the RBA algorithm is performed to estimate the variable step size of the duty ratio. It consists of a memory with a length of *N* bits. After the *N* bits are assigned, the aggregated value (*B*) is calculated. The architecture of the whole RBA block is drawn in Fig. 4.

Afterwards, the outputs of both the NN module ($P_{ref}$) and the RNA module (B) are used to calculate the variable step size ($\triangle {D}$) as indicated in the following equation:

4 $$\begin{aligned} \triangle {D} = (P_{old}- P_{ref})* d* B \end{aligned}$$

**Figure 5:**
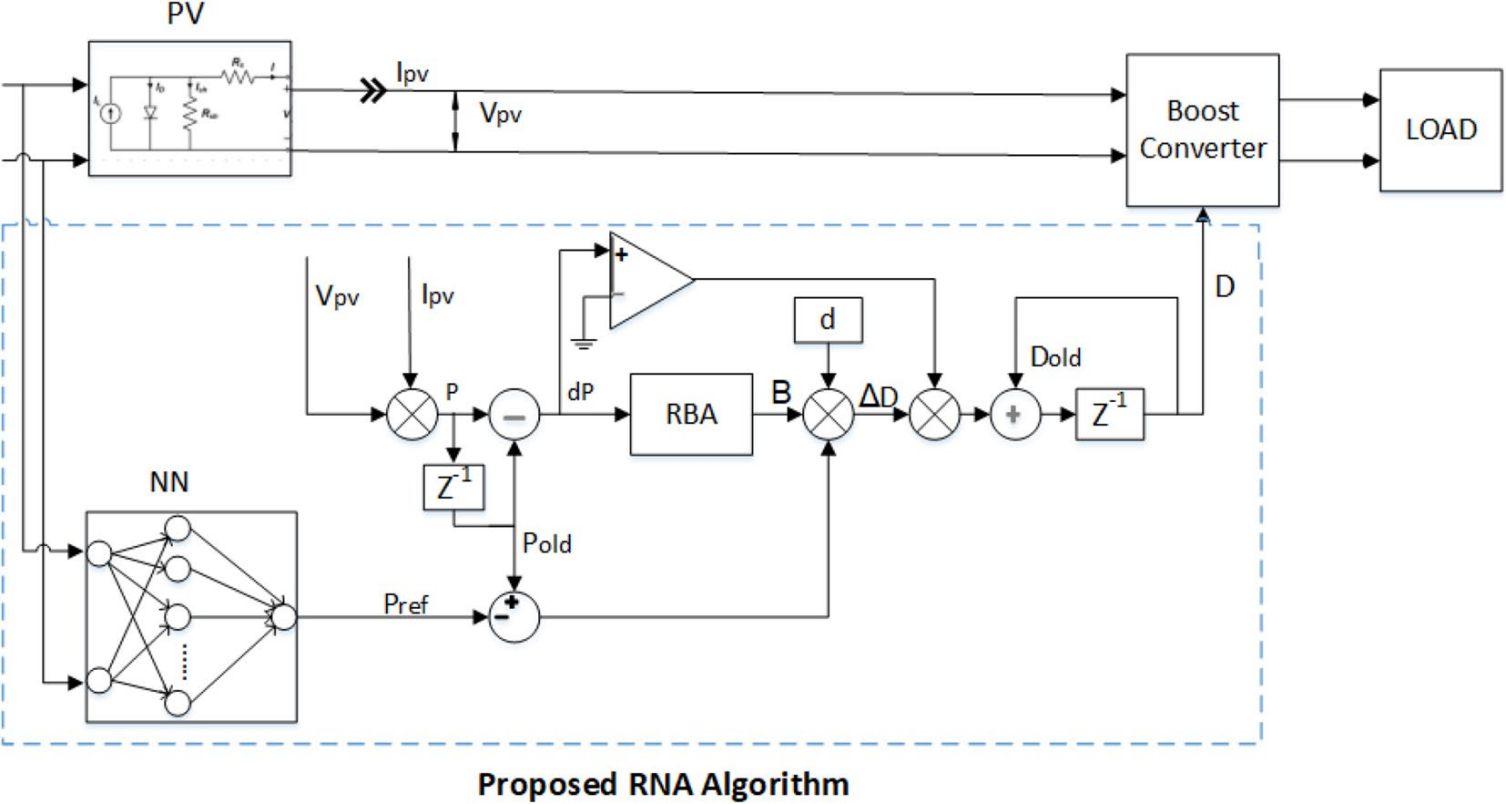
The proposed harvesting system architecture using RNA MPPT algorithm.

Finally, the value of the duty ratio (*D*) is calculated as

5 $$\begin{aligned} {D} = (D_{old}\, \acute{\hbox {s}} \,{\triangle {D}} ) \end{aligned}$$

where $D = (D_{old}+ \triangle {D})$ when $dP<0$. Otherwise, $D = (D_{old}- \triangle {D})$. This condition can be implemented using a comparator circuit. The hardware architecture of the proposed harvesting system using RBA algorithm is shown in Fig. 5.

### Recursive bit assignment (RBA)

As shown in Fig. 4, the RBA block consists of two comparators, X-NOR, memory with a size of $N*1$ bits, *N* multipliers, and one adder. First, *dP* is compared with zero. Then, the output of the comparator is X-nored with a rand comparator. The X-NOR circuit operation is in Table 1. Hence, the production output of the X-NOR circuit is stored successively in a sequential manner in the memory locations.

After all memory contents are assigned ($B_0$, ..., $B_{(N-1)}$), the output of the RBA block can be determined by summing the weighted values of the assigned bits, which can be written as:

6 $$\begin{aligned} B = \left[ \frac{B_{0}}{2^1}+ \frac{B_{1}}{2^2} + \cdots \frac{B_{N-1}}{2^N}\right] \end{aligned}$$

**Table 1:** The X-NOR circuit operation.

First comparator	Second comparator	X-NOR output
‘0’	‘0’	‘1’
‘0’	‘1’	‘0’
‘1’	‘0’	‘0’
‘1’	‘1’	‘1’

### Neural network based MPPT

This research uses a feed-forward neural network with one hidden layer of 10 neurons for the MPPT control design. It has two inputs (which are G and T) and one output ($P_{mpp}$) as shown in Fig. 5. The training process for the neural network is performed using the Levenberg-Marquardt backpropagation optimization method.

### DC–DC boost converter

**Figure 6:**
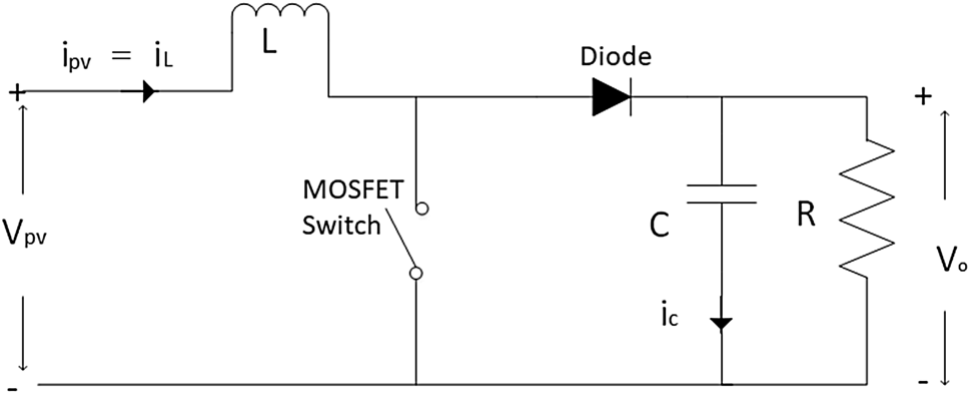
The DC–DC Boost converter.

**Figure 7:**
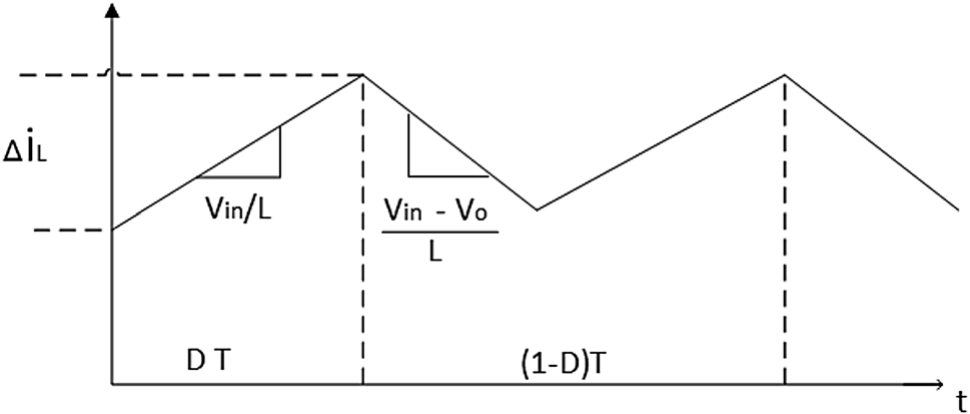
The inductor current waveform.

The DC-DC boost converter schematic diagram with a switching period of *T* and a duty cycle of *D* is shown in Fig. 6. It consists of an inductor with inductance *L*, capacitor *C*, a MOSFET switch, Diode, and a load *R*. When the switch is in on state, the inductor is charged from the input PV voltage $V_{pv}$, and the capacitor discharges across the load. From Fig. 7 (all figures from 1 to 7 are generated using Microsoft Visio professional-2019: https://www.microsoft.com/en-us/microsoft-365/p/visio-professional-2021/cfq7ttc0hgxw?activetab=pivot:overviewtab). The current ripple value in the inductor can be expressed as follows:

7 $$\begin{aligned} \triangle {i}_{L}= & {} {I}_{L}max - {I}_{L}min = \left( \frac{V_{PV}}{L}\right) * D {T} \end{aligned}$$

8 $$\begin{aligned} \triangle {i}_{L}= & {} \frac{({V}_{PV}*{D})}{L *({f}_{s})} \end{aligned}$$

where $\triangle {i}_{L}$ is the current ripple of inductor and must not exceed 30% of $I_L$, $I_L$ is the inductor current. Also ${I}_{L}max$ and ${I}_{L}min$ is the maximum and minimum value of the inductor current $I_L$. ${f}_{s}$ is the sampling frequency, D is the duty ratio of MOSFET.

Practically, the current ripple value is related by the choice of the ripple coefficient $K_{\triangle {i}}$:

9 $$\begin{aligned} K_{\triangle {i}} = \frac{\triangle {i_{L}}}{I_{L}} \end{aligned}$$

The inductance value of the inductor L can be calculated as:

10 $$\begin{aligned} L = \frac{V_{in}*D}{f_{s} *\triangle {i_{L}}} \end{aligned}$$

The capacitance filter (RC) can limit the ripple in the output voltage and provide a DC output current to the load when the switch is in off mode. The minimum value of capacitance should be:

11 $$\begin{aligned} C_{min} = \frac{D . {V_{o}}}{f_{s}.R . \triangle {V_{o}}} \end{aligned}$$

where $$ V_o$$ is the boost converter output voltage and $$\triangle {V_{o}}$$ is voltage ripple and must be 5% of the $$ V_o$$.

Finally, the transfer function of the DC-DC Boost converter in the s domain G(s) is:

12 $$\begin{aligned} G(S)= \frac{V_{o}}{1-D} * \frac{1- \frac{L S}{(1-D)^2 * R}}{\frac{LCS^2}{(1-D)^2}+\frac{LS}{(1-D)^2 R} + 1} \end{aligned}$$

## Performance metrics

The performance of the proposed algorithm can be measured using three parameters: algorithm efficiency, rise time, and ripple factor.

The algorithm efficiency can be defined as:

13 $$\begin{aligned} Efficiency = 1- \left[ \frac{(MPP_{algorithm} - MPP_{actual})}{MPP_{actual}}\right] * 100 \end{aligned}$$

where $MPP_{algorithm}$ is the obtained MPP by the proposed algorithm. $MPP_{actual}$ is the actaul value that can be obtained from the P–V characteristic curve.

Rise time can be defined as the time taken by the proposed system parameters to arrive steady-state value.

Also, the ripple around the MPP can be measured by “the ripple factor.” It can be defined as the difference between the maximum and the minimum power values divided by the minimum power value at the steady-state.The related power values are obtained from the output power curve of the proposed algorithm.

## Simulation analysis

### Simulation setup

**Figure 8:**
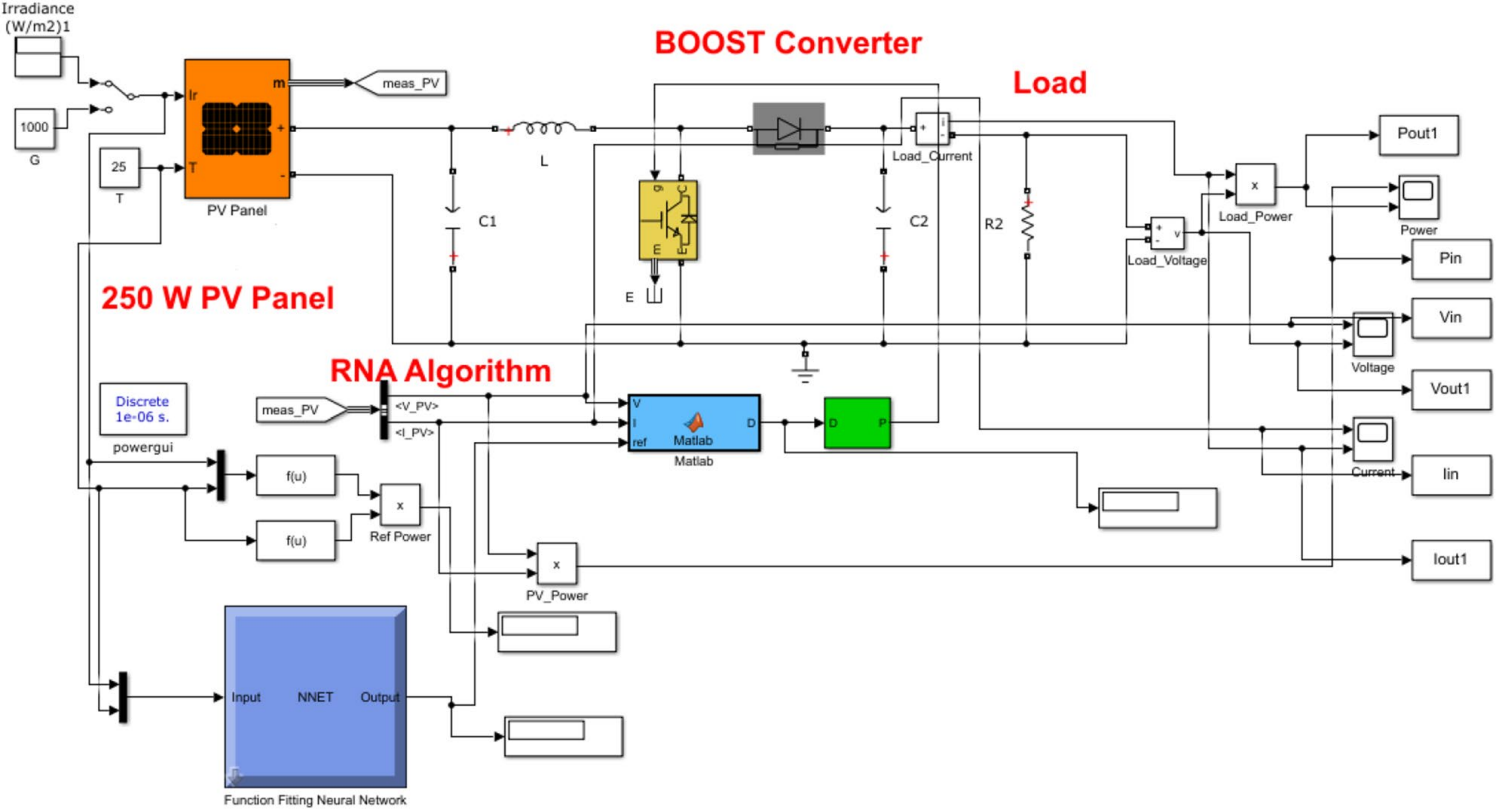
The overall Matlab Simulink structure of the proposed PV system.

The overall structure of the proposed PV system is designed using MATLAB Simulink as shown in Fig. 8. The proposed system uses a **Soltech 1STH-250-WH** PV module with a maximum power of 250W. The numerical parameter values for the PV module and the proposed RNA algorithm are listed Table 2.

**Table 2:** The simulation parameters.

Parameter	Value
The PV parameters	
Open circuit voltage, $V_{OC}$	37.3 V
Short circuit current, $I_{SC}$	8.66 A
Cells in a module, *N*	60
Ideality factor, *a*	1.019
Parallel Resistance, $R_{p}$	224.1886 $\Omega$
Series Resistance, $R_{s}$	0.23724 $\Omega$
The proposed RNA parameters	
Max duty ratio, $D_{max}$	0.8
Min duty ratio, $D_{min}$	0.08
Fixed step size, d	0.00001
Number of neural layers	10
Optimization algorithm	Levenberg–Marquardt (LM)
Memory size	N*1 bits
The boost converter parameters	
Inductance of inductor, *L*	$3 e^{-3}$ H
Conductance of capacitor, *C*	2000 ţF
Sampling frequency, $f_s$	10 KHz

### PV module characteristics

**Figure 9:**
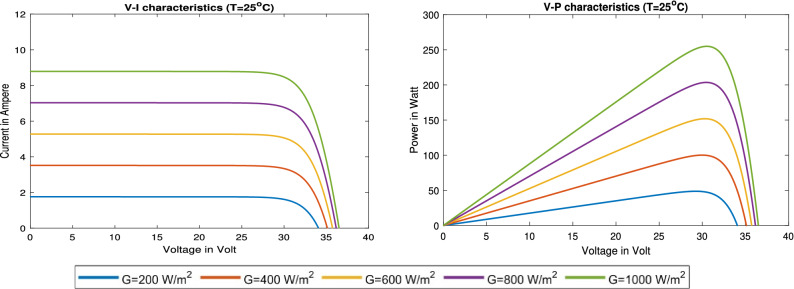
The V–I and V–P characteristic of the PV module.

The nonlinear P–V and I–V characteristics of the PV module for different irradiance values from 200 to 1000 W/m$^2$ are shown in Fig. 9. As shown, the maximum achievable power value for this type of PV is 250 *W* when irradiance and temperature values are 1000 W/m$^2$ and 25 $\,^{\circ} \text {C}$, respectively.

### Neural network performance

**Figure 10:**
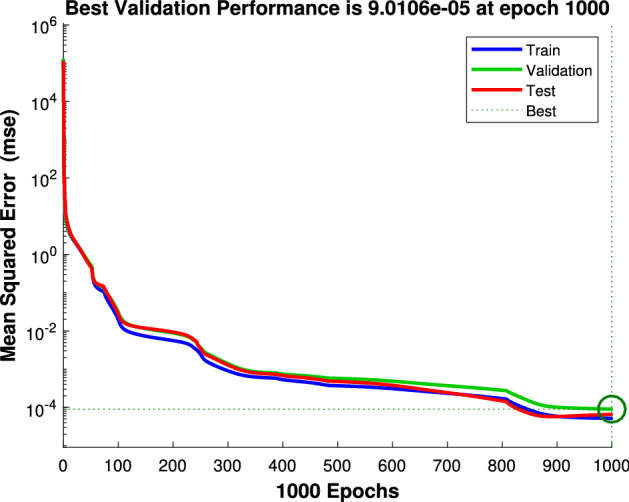
The performance of the neural network.

The designed NN module performance can be verified by the Mean Square Error (MSE) value. The MSE values of training, validation, and testing measurements within 1000 epochs are plotted in Fig. 10. From the results, the best validation performance is achieved at MSE of $9.0106\times {10^{-5}}$ at 1000 epochs. In addition, training, validation and testing curves are closed to each other. This means that the designed NN is reliable and can predict its output value efficiently.

### The proposed RNA algorithm performance

**Figure 11:**
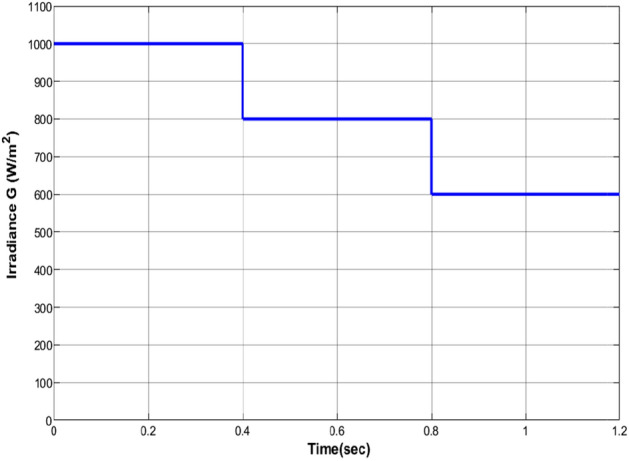
The input irradiance pattern.

Figure 11 plots the input irradiance pattern applied to the selected PV module at the room temperature of T= $25\,^{\circ}$C. It starts with 1000 W/m$^2$. After that, it is changed from 1000 to 800 and to 600 W/m$^2$ with a step time of 0.4 s.

**Figure 12:**
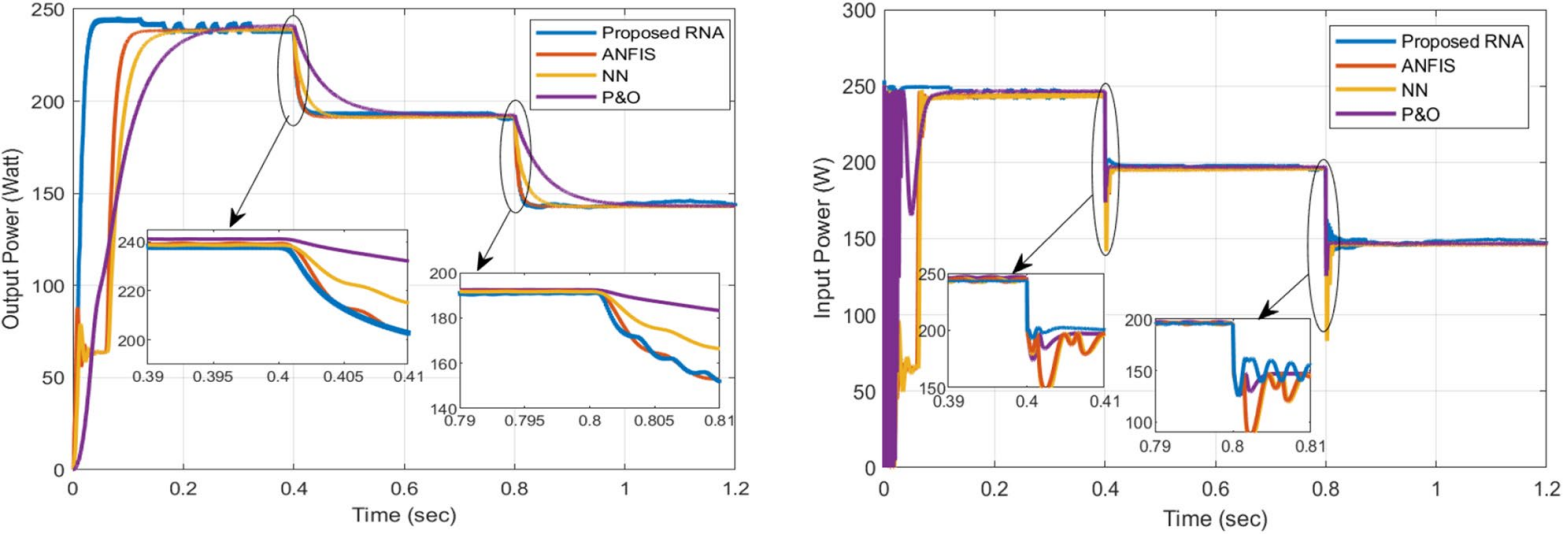
The output and input powers of the boost converter.

**Figure 13:**
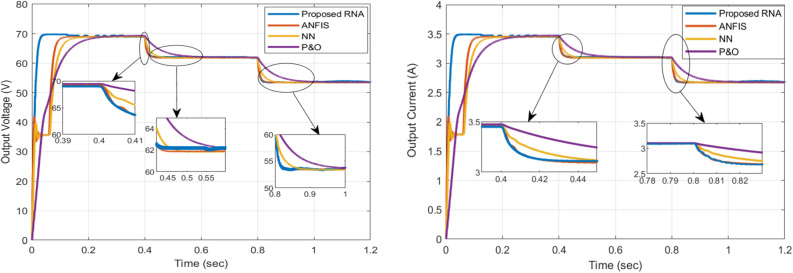
The output voltage and current of the boost converter.

The overall performance of the proposed RNA system is measured by three main parameters: achieving MPP, achieving high-speed MPP tracking, and reducing ripple around MPP. The boost converter output power, voltage and current curves are shown in Figs. 12 and 13. From the results, the proposed RNA system achieves the MPP with the fast-tracking response and acceptable oscillations around the MPP.

The figure on the left in Fig. 12 shows a comparison of the output powers of a DC-DC boost converter in the proposed RNA, ANFIS, NN and P &O algorithms. At G = 1000 W/m$^2$, 800 W/m$^2$, and 600 W/m$^2$, the proposed algorithm attains maximum output power of 245 W, 195 W, and 146 W, respectively with the fast-tracking response. In contrast, the NN-based and ANFIS algorithms have a slow response with less maximum power point values. At the same time, the conventional P &O provides the worst performance in terms of tracking response compared with all other algorithms. At G = 1000 W/m$^2$, the proposed system attains the maximum power value within 0.05 s. While, the ANFIS and NN-based algorithms arrive at a maximum power of 237 W and 236 W within 0.17 s and 0.12 s, respectively. From the results, the proposed RNA system achieves the best performance compared to other algorithms. Moreover, it also achieves the fastest response time with different irradiance conditions. Also, it has an acceptable ripple. The reason for this is the use of the variable step size RBA block that continues to adjust the duty ratio of the boost conversion. In addition, the performance can be improved by increasing the memory size of the RBA block.

The figure on the right in Fig. 12 shows the boost converter input power comparisons between the algorithms. Similarly, the best performance occurs with the proposed RNA system across all comparison metrics. At G = 1000 W/m$^2$, the proposed RNA achieves maximum power of 245 W at 0.06 s. At the same time, the other algorithms have more delays in reaching the MPP value. At G = 800 W/m$^2$, the ANFIS algorithm begins with a low PV power of 180 W at t = 0.4 s. It arrives at the MPP of 195 W at t = 0.44 s. The proposed algorithm approximately gets a stable power value of 194 W at t = 0.4 s. In comparison, the NN-based algorithm performs nearly the same response of ANFIS with power of 142 W at 0.4 s. In contrast, the P &O attains 173 W at t = 0.4 s and MPP of 198 W at 0.44 s.

The output voltages of the boost converter for the compared algorithms are shown in the left hand side in Fig. 13. For G = 1000 W/m$^2$, the proposed algorithm has the fastest response to arrive at the maximum output voltage at 70 V within 0.06 s. While the ANFIS requires about 0.14 s to get a maximum output voltage of 69 V. The NN-based algorithm needs 0.2 s to obtain the same performance as the ANFIS algorithm. Regarding the P &O, it has a slow response, as it needs more time of 0.3 s to follow up the same performance of the two previous algorithms.

The output currents for all compared algorithms are shown in the right hand side in Fig. 13. The proposed RNA reaches the maximum output current value of 3.5 A at a time of 0.06 s. At G = 800 ${\text{W/m}}^2$, the proposed RNA obtains the maximum output current value of 3.1 A at 0.4 s. The ANFIS algorithm has a faster response than the NN-based algorithm. While the P &O algorithm has the highest delay as it needs a time of 0.57 s to achieve the maximum value. Likewise, at G = 600 W/m$^2$, the proposed algorithm donates a maximum current of 2.7 A at 0.84 s. The NN-based algorithm needs 0.03 s to reach the same performance as the ANFIS algorithm. While the P &O algorithm requires 1 s to arrive at the maximum output current.

**Figure 14:**
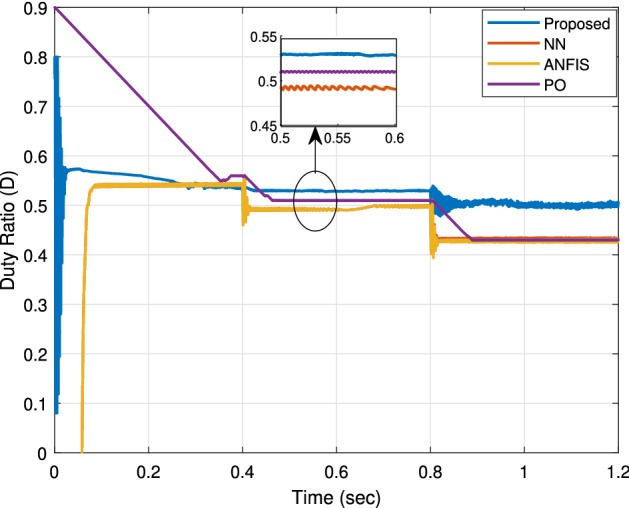
The duty cycle.

The duty cycle variation of the proposed RNA algorithm is compared with that of the other algorithms in Fig. 14. Changing the duty ratio is required to achieve the MPP value as the environmental condition of the PV module changes. At G = 1000 W/m$^2$, the proposed RNA algorithm gets the same duty ratio of 0.55 approximately all the period time of 0.4 s. The NN and ANFIS algorithmS have a high impulses duty ratio at each transition point. In addition, the proposed attains the stable duty ratio after 0.0338 s. While the two algorithms(NN and ANFIS) attain the same value after approximately 0.23 s. At G = 800 W/m$^2$, the proposed RNA algorithm achieves a duty ratio of 0.53 while, the NN and ANFIS algorithms attain a duty ratio of 0.53 with a noticeable ripple. This explains the stable output performance of the boost converter in the proposed RNA system. The P &O algorithm draws a decreasing duty cycle curve. At G = 8000 W/m$^2$, it donates a duty cycle value of 0.51 at a time of 0.46 s.

The summary of the comparisons between the proposed RNA algorithm and other related MPPT control algorithms with different irradiance levels (1000, 800, 600 W/m$^2$) are listed in Table 3. The most important metrics are the rise time (the fast tacking response), the algorithm efficiency, and the ripple factor.

**Table 3:** Summary of comparisons of different MPPT control algorithms.

Parameter	Irradiance	Output voltage	Output power	MPP algorithm	Actual MPP	Rise time	Efficiency (eq. 13)	Ripple factor
	W/m$^{2}$	*V*	Watt	Watt	Watt	s	%	
Proposed	1000	69.76	244	247.4	250	0.052	98.96%	0.436
	800	62.3	194.3	197.87	200	0.43	98.9%	0.53
	600	54	145.6	148.92	150	0.87	99.28%	0.52
ANFIS	1000	69	238.7	244.62	250	0.13	97.84%	0.202
	800	61.9	192.7	196.65	200	0.45	98.3%	0.08
	600	53.5	143.07	146.5	150	0.85	97.66%	0.05
NN	1000	68.9	238	244	250	0.18	97.6%	0.062
	800	61.8	191.27	196.4	200	0.489	98.2%	0.03
	600	53.4	142.75	145.95	150	0.892	97.3%	0.01
P &O	1000	69.1	240	246	250	0.3	98.4%	0.027
	800	62	192.8	197	200	0.6	98.4%	0.01
	600	53.5	143.2	146.7	150	1	97.8%	0.01

###  The impact of varying the irradiance profile

**Figure 15:**
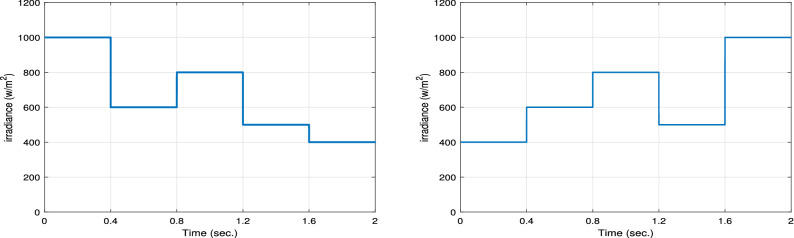
Two different irradiance profiles G1 (left) and G2 (right).

In this section, the effect of changing irradiance profiles is investigated at the same room temperature ($\text {T} = 25\,^{\circ }\text {C})$. Two different input irradiance profiles are applied to the proposed RNA system as shown in Fig. 15. The left hand side in Fig. 15 indicates the irradiance profile (G1) while the right hand side one indicated the irradiance profile (G2). As shown, the transitions of G1 every 0.4 s are from 1000 to 600 to 800 to 500 to 400 W/m$^2$. While, the transitions of G2 range from 400 to 600 to 800 to 500 to 1000 W/m$^2$.

**Figure 16:**
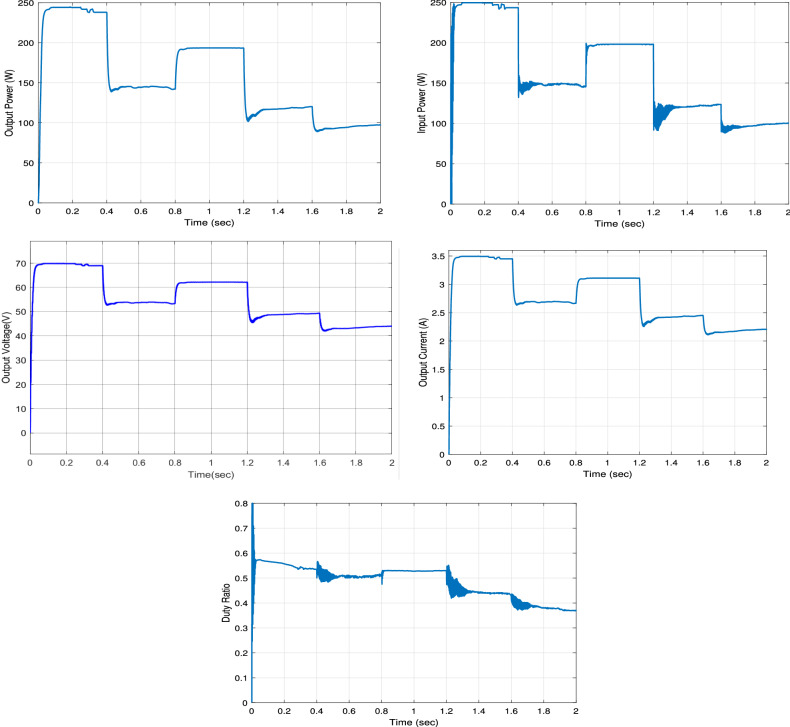
The output power, input power, output voltage, output current and the duty ratio under the first irradiance profile G1.

Figure 16 plots the output power, input power, output voltage, output current, and the duty ratio of the boost converter with the proposed RNA algorithm under the application of the first irradiance profile G1. At G = 1 KW/m$^2$, the proposed RNA system gives a maximum output power of 245 W and a maximum output voltage of 70 V and a maximum current of 3.5 A. The input power curve gets a maximum value of 250 W which is the maximum power of that PV module type at the same G value. Then, the proposed RNA achieves an output power of 195 W and 121 W with a maximum output voltage of 62 V and 49 V and a maximum current of 3.1 A and 2.46 A at G =800 and 500 W/m$^2$, respectively. The duty cycle curve of the proposed RNA algorithm under the first irradiance profile G1 is drawn in Fig. 16. The duty cycle is plotted with the time under variable values of irradiances. At G= 0.6 KW/m$^2$ and at G= 0.8 KW/m$^2$, the proposed RNA algorithm can donates a maximum duty ratio of 0.51 and 0.52, respectively.

**Figure 17:**
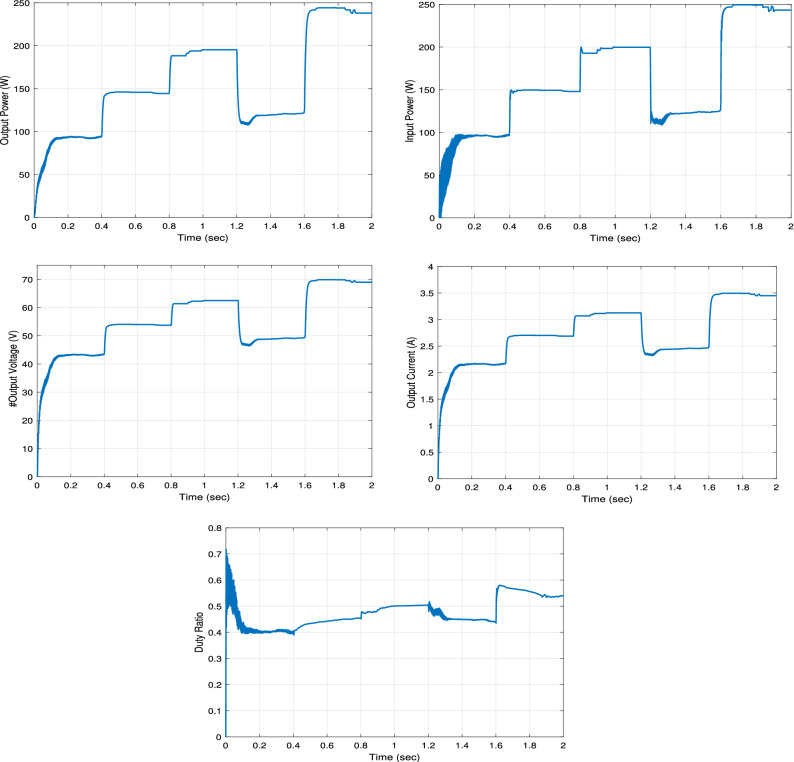
The output power, input power, output voltage, output current and the duty ratio under the second irradiance profile G2.

The output power, the input power, the output voltage, the output current, and the duty ratio of the boost converter using the proposed RNA algorithm under applying the second irradiance level G2 are drawn in Fig. 17. As shown, at G= 400 W/m$^2$ and 600 W/m$^2$, the proposed RNA attains a maximum output power of 95 W and 144 W, respectively. Further, it achieves a maximum output voltage of 44 V and 54 V, respectively at the same G values. The maximum input powers of the proposed RNA are 150, 200, 123 W at G= 600, 800, and 500 W/m$^2$, respectively. Also, the maximum output currents are 2.1, 3.1, and 2.4 A at G= 400, 800, and 500 W/m$^2$, respectively. The duty ratio of the proposed RNA in the second pattern is drawn with the time. It donates an increasing curve with a maximum values of 0.5 at G= 800 W/m$^2$ and a decreasing curve with a value of 0.45 at a time of 1.4 s for G= 500 W/m$^2$, respectively.

### The impact of varying the sampling time

**Figure 18:**
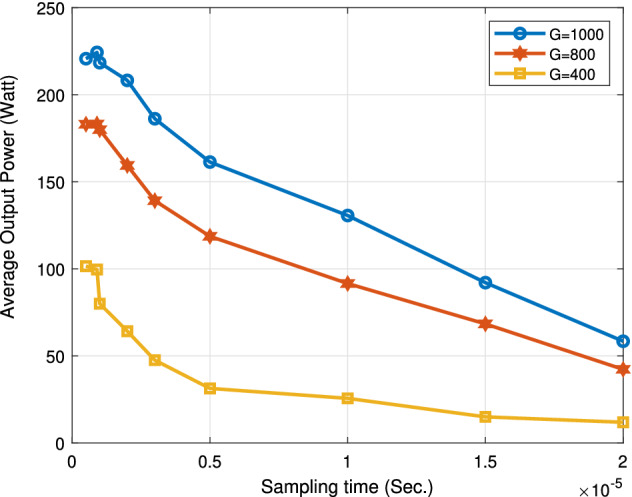
Effect of changing sampling time on average output power.

**Figure 19:**
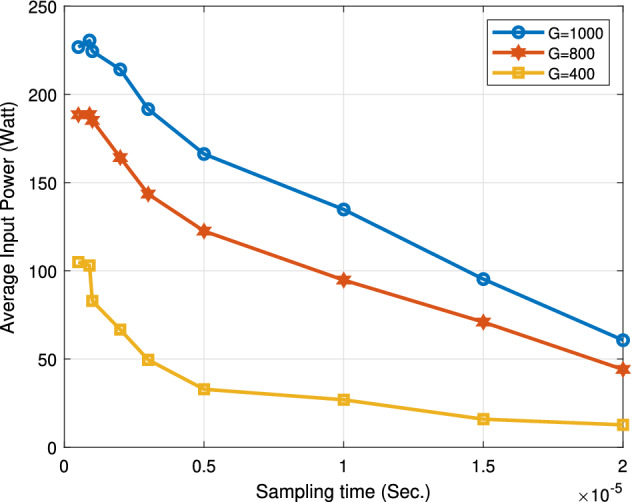
Effect of changing sampling time on boost converter average input power.

**Figure 20:**
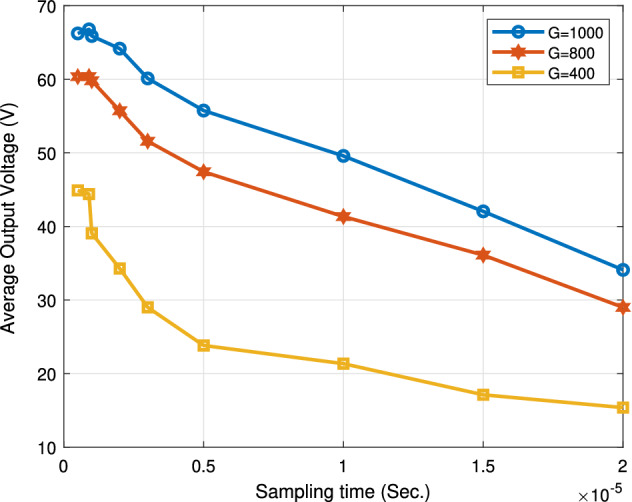
Effect of changing sampling time on average output voltage.

**Figure 21:**
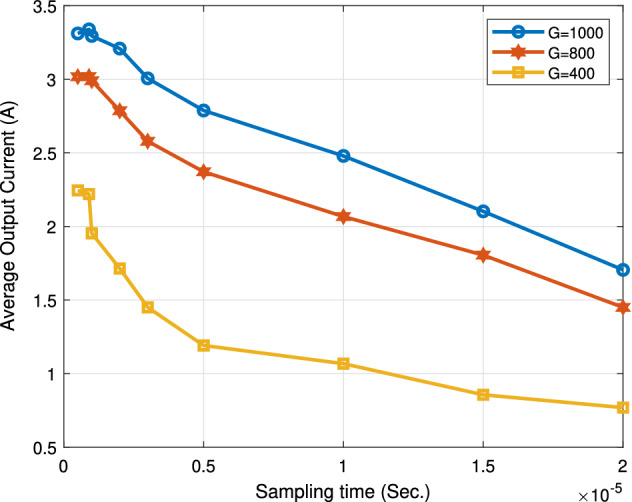
Effect of changing sampling time on average output current.

The following Figs. 18, 19, 20 and 21 study the impact of changing the sampling time on the performance of the proposed system. This impact appears on the boost converter output power, the PV power, the boost converter output current, and the output voltage. From the results, the value of the sampling time that donates the best performance of the proposed algorithm must not exceed 1 ţs. Consequentially, the sampling frequency is about 1000 KHZ. Increasing the sampling time by more than 1 ţs will result in an obvious degradation in the system performance. The optimum range value of the sampling time is from 0.1 to 1 ţs. Thus, the sampling frequency range is from 1000 KHZ to 10 MHZ.

Figure 18 sketches the average output power with the sampling time for three different irradiance levels (G = 1000 W/m$^2$, G = 800 W/m$^2$, G = 400 W/m$^2$). The maximum average output power is achieved at the sampling time range from 0.1 to 1 ţs. The proposed system suffers from a large degradation in its performance for the sampling time over 1 ţs. At G = 1000 W/m$^2$ and sampling time of 5 ţs, the proposed system donates an average output power value of 162 W, which is away from the maximum mean output power.

Similarly, the effect of varying the sampling time on the boost converter average input power is drawn in Fig. 19. At a sampling time of 10 ţs and G = 800 W/m$^2$, the proposed algorithm attains average input power of 194.7 W.

Figure 20 draws the effect of varying the sampling time on the boost converter average output voltage. Similarly, The sampling time should be equal to or less than 1 ţs. As a sequential, the sampling frequency must be greater than or equal to 1000 KHZ. The optimum value of the sampling time is 1 ţs, at which the proposed algorithm gets a maximum mean output voltage of 65.8 V, 60 V and 44 V at G = 1000 W/m$^2$, G = 800 W/m$^2$ and G = 400 W/m$^2$, respectively.

Figure 21 explains the effect of changing the sampling time on the boost converter output current. The average output current scores a maximum values of 3.3 A, 2.99 A, and 2.2 A at G = 1000 W/m$^2$ , G = 800 W/m$^2$, and G = 400 W/m$^2$, respectively.

### The impact of severe irradiance and temperature conditions

**Figure 22:**
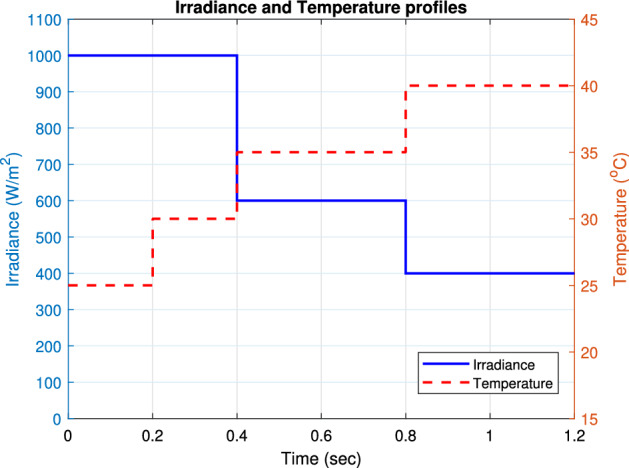
Irradiance and temperature profiles.

The proposed RNA algorithm is operating under fast temperature (T) changes and different solar irradiance conditions (G) at the same time. Figure 22 sketches the temperature profile and solar irradiance profile in which the proposed can be applied. The temperature profile consists of four different values of temperature ($25\,^\circ$C, $30\,^{\circ}$C, $35\,^{\circ}$C, $40\,^{\circ}$C) in which the proposed RNA algorithm operates at a different temperature under a defined irradiance level of G. At room temperature T = $25\,^{\circ}$C and at T = $30\,^{\circ}$C, the proposed RNA is exposed to an amount of radiation of 1000 W/m$^2$. Also, it is exposed to solar radiation of 600 and 400 W/m$^2$ at T = $35^{\circ }$C and $40^{\circ }$C, respectively.

**Figure 23:**
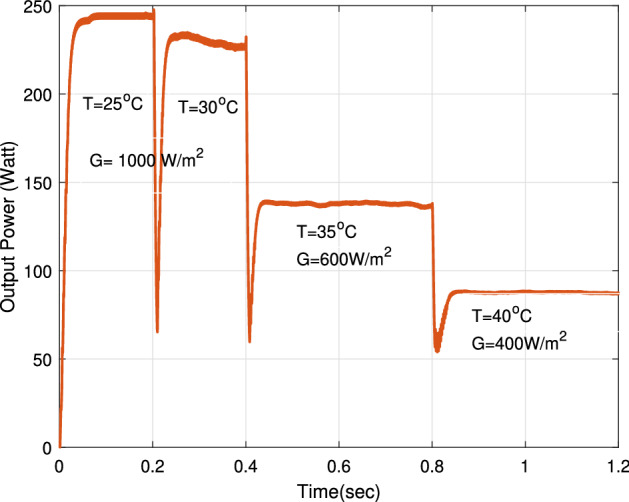
The impact of fast temperature changes on the output power of proposed RNA algorithm under different irradiance levels.

The output power of the proposed RNA algorithm under fast temperature changes and different irradiance levels at the same time is drawn in Fig. 23. At G= 1000 W/m$^2$, the proposed technique remains obtaining the maximum value of 245 W at T = $25^{\circ }$C. But, the performance of the proposed degrades with increasing the temperature. At T = $30^{\circ }$C, the proposed scores a maximum value of 235 W. The effect of changing temperature is highly affects the high levels of irradiance of G. At G = 600 W/m$^2$, 400 W/m$^2$, the proposed attains a maximum value of 137, and 88 W at T = $35\,^{\circ}$C, T = $40\,^{\circ}$C, respectively. The impact of fast temperature changes degrade the performance of the proposed hardly at the beginning of the transitions. Furthermore, it gets back up quickly to a certain value with a steady state response.

**Figure 24:**
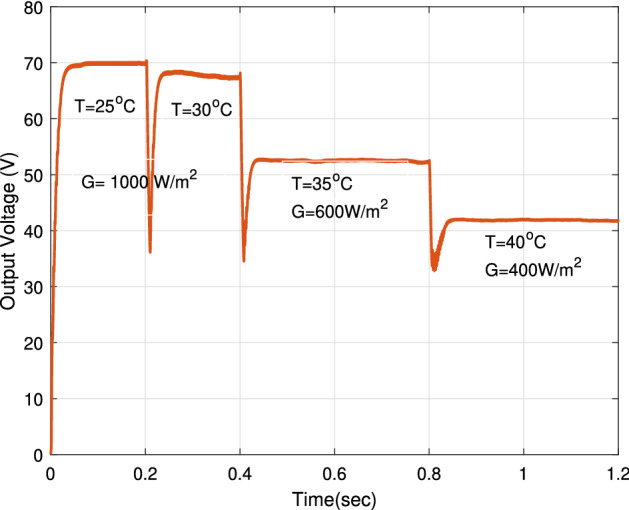
The impact of fast temperature changes on the output voltage of proposed RNA algorithm under different irradiance levels.

The output voltage of the proposed RNA algorithm under fast temperature changes with the time is indicated in Fig. 24. With increasing temperature degrees, a drop off has been done at the beginning of each curve at each different irradiance level. After that, the proposed RNA still keeping a steady state response. At T = $30\,^{\circ}$C, the proposed donates a maximum value of 68 V. At T = $35\,^{\circ}$C, T = $40\,^{\circ}$C, the proposed gets a maximum value of 52 V and 41 V at G = 600 W/m$^2$, 400 W/m$^2$, respectively.

### The performance under partial shading condition (PSC)

Partial shading is a problem that occurred in the PV system. It is caused by the shading effect on some photovoltaic cells. Therefore, some cells will be partially or completely closed. Thus, the power generated by the PV module will be lower than its normal value. Moreover, the PV characteristics curve will have more than one power peak (local peak per shaded part). But, the maximum power with a high peak is chosen to be the Global Maximum Power Point (GMPP). Hence, the mission of the MPPT system is to track global maximum power point.

**Figure 25:**
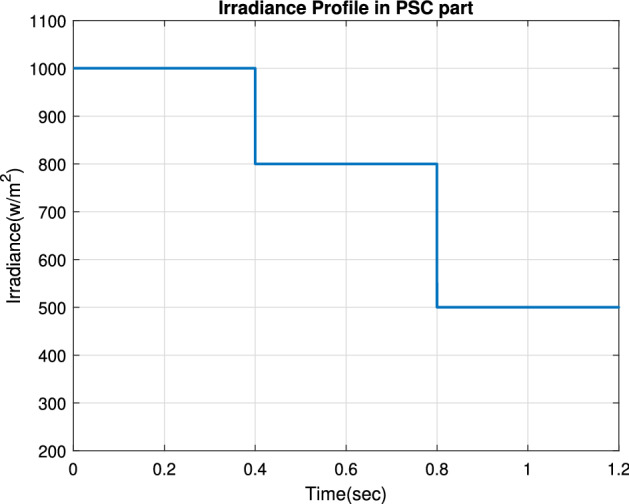
The irradiance profile in PSCs.

In this subsection, we examine the effect of applying partial shading conditions (PSCs) to PV on the performance of our proposed RNA algorithm. The PV array is built from parallel strings, each with series connected modules. Thus, there are multiple local maximum power points (LMPPs) in the PV characteristic curve of the photovoltaic array. Assuming that the initially irradiance profile applied as shown in Fig. 25 varies from G = 1000 to 800 to 500 W/m$^2$, respectively at room temperature (T = $25\,^{\circ}$C). In addition, two PSCs scenarios are tested, namely pattern A scenario and pattern B scenario.

**Figure 26:**
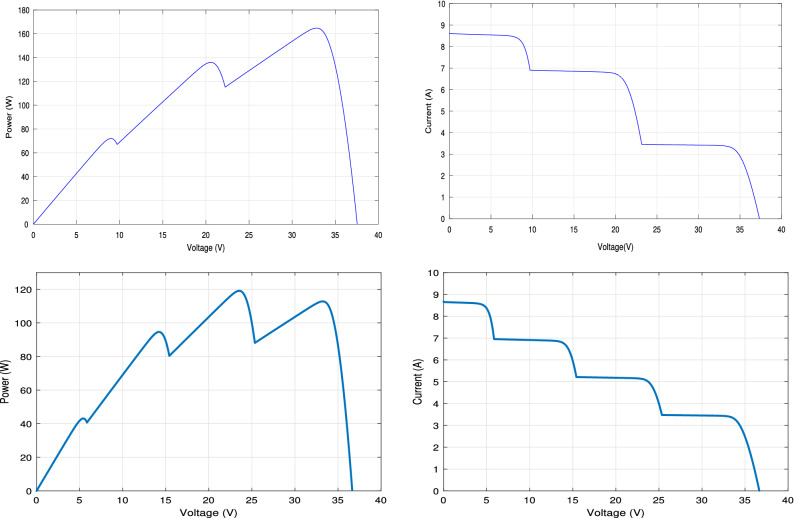
The P–V and I–V characteristics curves of the PV array Under PSC in the two patterns A and B scenarios.

**In pattern A scenario**, the PV array composes of three parallel strings, each string contains 20 series connected cells. So, there is three LMPPs in its PV characteristics curve. The first string with cells from 1 to 20 is applied at full irradiance level (100%). The second strings (from cell number 21–40) is applied at 80% of full irradiance level. While, 40% of full irradiance level is applied to the third string (from cell 41 to 60). The P–V and I–V characteristics curves of PV array under PSCs in pattern A scenario are drawn in the left side of Fig. 26.

**In pattern B scenario**, the PV array is modeled as four parallel strings, each string contains 15 series connected cells. Thus, four LMPPs are located in the PV characteristic curve. The first string with cells from 1 to 15 is applied at full irradiance level. While, 80%, 60% and 40% of full irradiance level are applied to the second, the third and the fourth string, respectively. The P–V and I–V characteristics curves of PV array under PSCs in pattern A scenario are drawn in the right side of Fig. 26.

**Figure 27:**
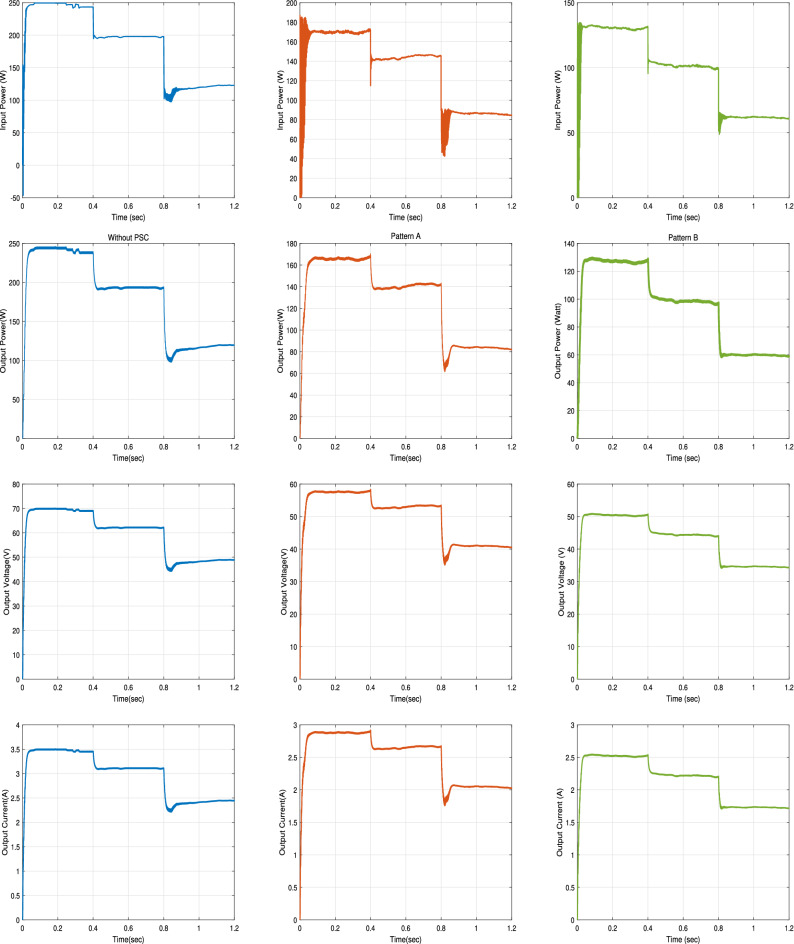
Input power, output power, output voltage, and output current of the Proposed RNA algorithm without, with PSCs of pattern A, and with PSCs of pattern B scenarios (from left to right, respectively).

**Figure 28:**
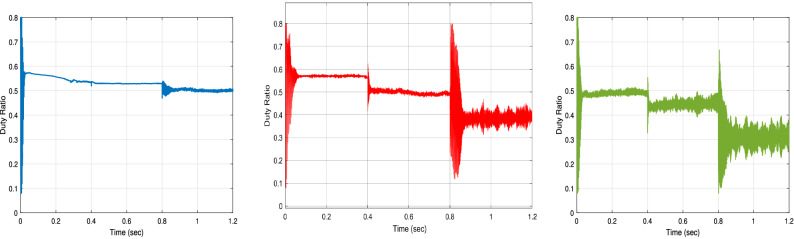
The duty ratio of the Proposed RNA algorithm without, with PSCs of pattern A, and with PSCs of pattern B scenarios (from left to right, respectively).

The input power, the output power, the output voltage, and the output current curves of the proposed RNA algorithm with and without PSCs in the two patterns A and B scenarios are exposed in Fig. 27. While, the duty ratio curves are plotted in Fig. 28. The performance is simulated at room temperature of T = $25\,^{\circ}$C with applied irradiance plotted in Fig. 25. From results, the maximum output power at each irradiance level is reduced under PSCs as the PV curve exhibits multiple maximum peaks. The proposed system attains a maximum output power values of 245 W, 194 W and 120 W, at G = 1000, 800 and 500 ${\text{W/m}}^2$, respectively without any PSCs. While, it scores a maximum input power values of 250, 199, and 123 W, respectively. As well, it attains a maximum output power of 167 W, 140 W and 85 W and a maximum input powers of 170, 145, and 86 W under PSCs in pattern A and with the same irradiance levels as above. The proposed algorithm under pattern B gets a lower output power than pattern A due to the increase in the number of LMPPs. At the same values of G values, the proposed system records maximum output powers of 126 W, 100 W and 60 W, respectively, in pattern B. The maximum input power values of the proposed algorithm in the pattern B are 130, 102, and 63 W, respectively.

The output voltage of the proposed RNA algorithm attains higher values without PSCs than the PSCs effect as shown in Fig. 27. Under PSCs in pattern A, the output voltage is decreased from a maximum value of 70 V to 57.5 V at G = 1000 W/m$^2$. Also, the maximum output voltage records 62 V and 49 V for G = 800 and 500 W/m$^2$, respectively without PSC. These values drop to 53 V and 42 V at the same values of G, respectively under PSC in pattern A. The proposed algorithm gets a maximum voltage values of  50 V, 44.5 V, and 33.6 V at the same values of G = 1000, 800 and 500 W/m$^2$, respectively.

Also, the output current of the proposed RNA algorithm donates the best performance without PSC as shown in Fig. 27. At time 0.2 s, the proposed gives a maximum output current of 3.5 A, 2.86 A, and 2.5 A without PSCs and under PSCs in the two patterns (A and B), respectively. This is due to the proportional relation between the current flow and the irradiance levels in each module or string. At G = 800 and 500 W/m$^2$, the proposed algorithm attains a decreasing current values of 2.6 A and 2 A under PSCs in pattern A and of 2.2 A and  1.7 A for pattern B, respectively.

The duty cycles curves of the proposed RNA algorithm without any shading is compared with that of the proposed algorithm under the two patterns (A and B) as shown in Fig. 28. The proposed donates a maximum duty ratio of 0.57 at G = 1000 W/m$^2$ with a smooth response. The proposed gets a duty ratio curve with a high ripples with applying the shading effects conditions. Also, the reduction of the duty ratio values has been obtained with PSCs (pattern A and B). At time = 0.6 s, the proposed donates a duty ratio of 0.53 without any pscs and 0.49 with a slightly ripple for pattern A. it gives a duty ratio of 0.44 with a high ripple at the same time of 0.6 s for pattern B.

**Figure 29:**
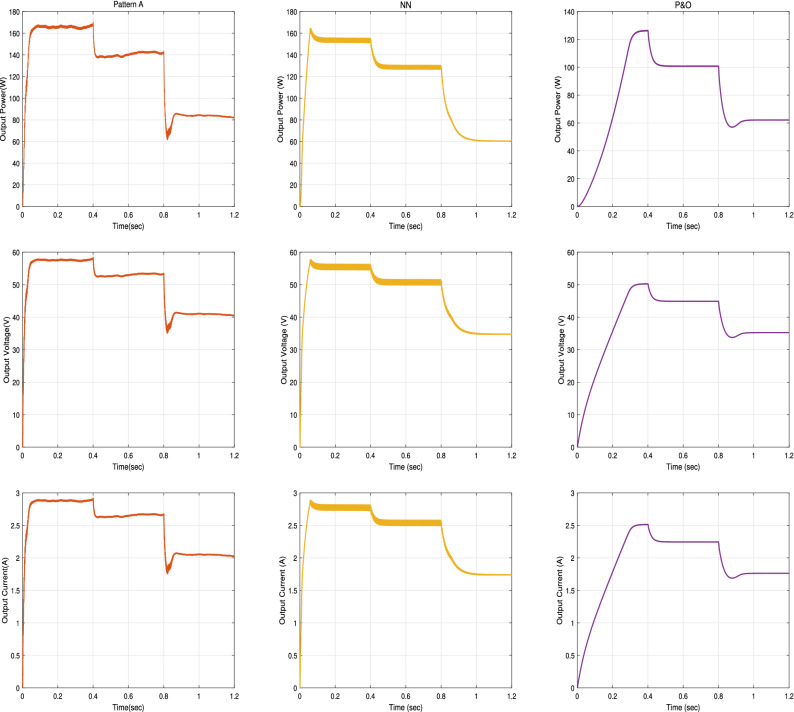
The output power, voltage and current of the Proposed RNA algorithm and other traditional algorithms under PSCs in pattern A.

Furthermore, the proposed RNA algorithm is compared with other traditional algorithms like NN and P &O algorithms with pattern A PSCs scenario under the same irradiance and temperature conditions as shown in Fig. 29.

As shown, the proposed RNA still performs best even under PSCs. At G = 1000 W/m$^2$, the proposed RNA achieves the highest output power of 167 W. NN obtains a stable maximum power value of 155 W at a time of 0.1 s at the same value of G. P &O gets a maximum output power of 125 W at a time of 0.33 s. At G = 800, 500 W/m$^2$, the traditional algorithms record a maximum power of 127, 60 W for NN and 100, 62 W for P &O, respectively.

The maximum output voltages of NN algorithm are 55, 50, and 35 V for G = 1000, 800, and 500 W/m$^2$, respectively. Also, P &O algorithm donates maximum values of 50, 44, 35 V at the same values of G, respectively.

For the output current performance, at G = 1000 ${\text{W/m}}^2$, the proposed in pattern A attains a maximum current value of 2.88  A at a time of 0.06 s. While, NN and P &O reach maximum values of 2.77 A and 2.5 A at times of 0.1 and 0.3 s, respectively at the same value of G. The traditional algorithms record maximum output currents of 2.6 A, and 1.75 A for NN and of 2.2, and 1.7 A for P &O at G = 800, and 500 W/m$^2$, respectively.

**Figure 30:**
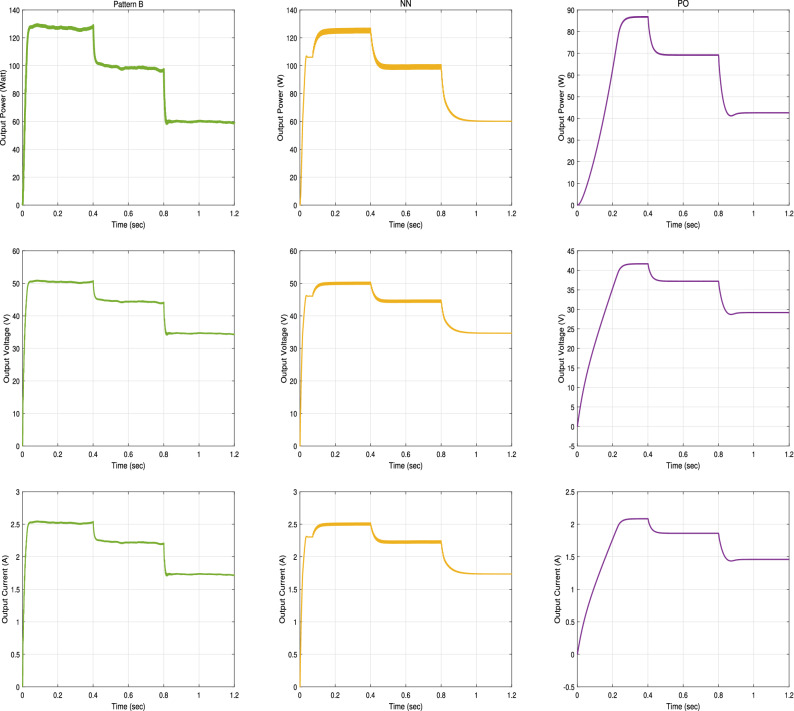
The output power, voltage and current of the Proposed RNA algorithm and other traditional algorithms under PSCs in pattern B.

Moreover, comparisons between the proposed RNA algorithm in the second PSCs pattern B scenario and other traditional algorithms (NN, P &O) under the same PSCs conditions are shown in Fig. 30.

The proposed RNA algorithm obtains the highest output power of 128 W at 0.03 s in the pattern B at G = 1000 W/m$^2$. NN can donate a maximum output power of 126 W at 0.14 s with a high ripple at the same G value. P &O attains a maximum output power of 86.8 W with a delay of 0.3 s. The proposed RNA algorithm achieves a maximum output power of 100 and 60 W at G = 800, 500 W/m$^2$ under PSC in pattern B, respectively. At G = 800 W/m$^2$, NN reaches a maximum output power ranging from 98.1 to 100 W at the same time of 0.6 s with a slow response. P &O gives a maximum output power of 69 and 43 W at G = 800, 500 W/m$^2$, respectively.

The maximum output voltages of NN system are 50, 45, and 35 V with a high ripple at at G = 1000, 800, 500 W/m$^2$, respectively. Also, P &O gets a maximum output voltages of 42, 37, and 30 V at the same G values. Furthermore, the maximum output current of NN system are 2.5, 2.2, and 1.7 A at the same G values. P &O donates a maximum current values of 2.1, 1.8, and 1.4 A, respectively. Finally, a summary of the results comparison between the proposed RNA algorithm and other related research is shown in Table 4.

**Table 4:** Comparison of the proposed algorithm with other related researches.

Algorithms	Results
Proposed RNA algorithm	Max $P_{out} = 247.4$ W, a fast rise time of 0.052 s, $\eta =98.9\%$
Proposed RNA algorithm under PSC-pattern A	Max $P_{out} = 167$ W, a rise time of 0.07 s, $\eta =98.06\%$
Proposed RNA algorithm under PSC-pattern B	Max $P_{out} = 129.7~W$, a rise time of 0.09 s, $\eta =98.48\%$
MPPT of PV under partail shaded through a colony of fireflies^12^	$\eta = 99\%$, with a tracking speed of 2.1 s under PSC
Comparison between neural network and P &O method for PV cell^19^	Pin=196 W for G = 1000 W/m$^2$, and 200 W PV, $\eta =98\%$ with fast tracking time
Neural network approach to MPPT control and irradiance estimation^26^	Track MPP with an error of 0.001% with a faster convergence speed
ANFIS current voltage controlled MPPT algorithm^27^	Pin = 247 W for G = 1000 W/m$^2$, $\eta =98.8\%$, Rise time of 0.3 s
SAR Algorithm method in PV system using MPPT^28^	The proposed raised the MPP efficiency from 61.7 to 80.85% with less energy loss

##  Experimental validation

**Figure 31:**
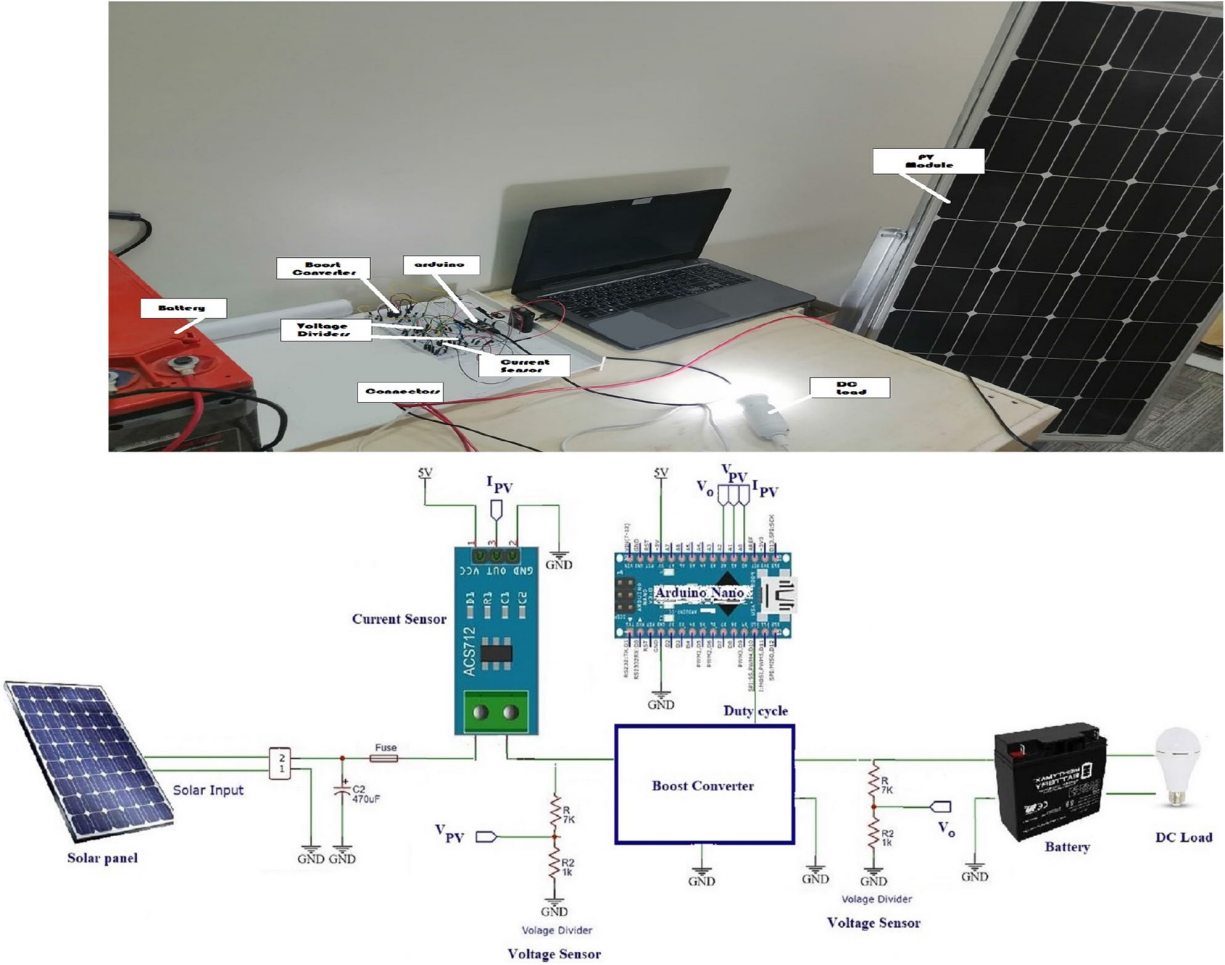
Experimental setup.

The prototype of the entire proposed MPPT controller system is shown in Fig. 31 (the figure is generated using Microsoft Paint application: https://apps.microsoft.com/store/detail/paint/9PCFS5B6T72H?hl=en-us &gl=us). The experimental circuit consists of the following main parts; the PV panel, the boost converter (and the driver circuit), the battery, the DC load, respectively. The boost converter is driven by Arduino nano micro-controller which is programmed with the proposed MPPT controller using Arduino IDE software.

For cost reasons, current and voltage sensors are used instead of irradiance and temperature sensors to be used as inputs for the RNA algorithm. The voltage sensor can be implemented by using a simple voltage divider circuit. While, ACS712 current sensor is used to measure the PV current.

**Figure 32:**
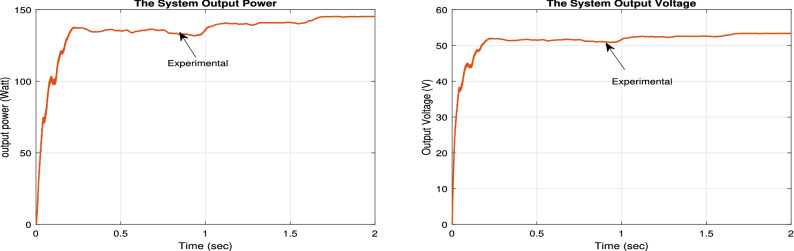
The experimental results.

The PV solar panel used is 250 W (with open circuit voltage of 30.7 v and maximum current of 8.15 A) solar panel. The capacitor($C_{2}$ = 470 ţF) is used at the output of the PV panel to remove any unwanted noise signal. The current from the PV panel is sensed using the current sensor ACS712 (which can sense up to 20 A with sensitivity of 100 mV/A). Then, this current value is input into the Arduino. Also, the voltage form the PV panel is sensed using voltage divider circuit and its output is fed to the Arduino. The used 12 v battery for storing the power has $V_{max}$ = 14.8 v, and charging current of 2.1 A. To measure the charging voltage, a voltage divider circuit is used. Also, its reading value is fed to the Arduino circuit.The voltage divider circuits are implemented by two resistors ($R_{1}$ and $R_{2}$) voltage divider circuit. $R_{1}$ = 7 K$\Omega$ and $R_{2}$ = 1 K$\Omega$. Finally, the DC load is selected as a 12 v DC lamp. The experimental results for the proposed system are shown in Fig. 32.

## Conclusions

In this paper, the proposed RNA MPPT controller algorithm for the solar PV system has been introduced. It has consisted of two main stages: the NN stage and the RBA algorithm stage. In the NN stage, the reference PV power can be obtained. In the RBA stage, the variable step of the duty cycle for the boost converter has been provided. Many comparisons between the proposed RNA algorithm and other related techniques such as; ANFIS, NN-based, and P &O have been executed. From the simulation results, the proposed RNA system has achieved superior performance in terms of the MPP’s fast-tracking and efficiency. But it has done a little higher ripple compared to other algorithms. In addition, the effect of varying the sampling time and varying the irradiance profile have been simulated. Moreover, the performance under PSCs has been verified. Further, the performances of two PSC patterns have been explained. Besides, the effect of the severe irradiance and temperature conditions have been tested. Finally, a small experimental verification of the proposed system has been presented.

The future work of our research will be the detailed experimental validation of an automatic battery charging controller circuit based on efficient MPPT algorithm. In addition, protection techniques will be concerned to provide the suitable circuit protection. Moreover, monitoring techniques may be introduced to maintain the status of the battery.

## Data Availability

All data sets in this paper are normally available for publishing. Also, the data that support the findings of this paper are available from the corresponding author upon reasonable request. Moreover, the data are not publicly available due to privacy.
